# Dental Applications of Carbon Nanotubes

**DOI:** 10.3390/molecules26154423

**Published:** 2021-07-22

**Authors:** Marco A. Castro-Rojas, Yadira I. Vega-Cantu, Geoffrey A. Cordell, Aida Rodriguez-Garcia

**Affiliations:** 1 Tecnologico de Monterrey, Escuela de Ingeniería y Ciencias, Ave. Eugenio Garza Sada 2501, Monterrey 64849, NL, Mexico; A00232038@itesm.mx; 2 Natural Products Inc., Evanston, IL 60202, USA; pharmacog@gmail.com; 3 Department of Pharmaceutics, College of Pharmacy, University of Florida, Gainesville, FL 32610, USA; 4 Universidad Autónoma de Nuevo León, Facultad de Ciencias Biológicas, Instituto de Biotecnología, San Nicolás de los Garza 66455, NL, Mexico

**Keywords:** carbon nanotubes, glass ionomer cements, dental restorations, tissue engineering, drug delivery systems

## Abstract

Glass ionomer cements and resin-based composites are promising materials in restorative dentistry. However, their limited mechanical properties and the risk of bulk/marginal fracture compromise their lifespan. Intensive research has been conducted to understand and develop new materials that can mimic the functional behavior of the oral cavity. Nanotechnological approaches have emerged to treat oral infections and become a part of scaffolds for tissue regeneration. Carbon nanotubes are promising materials to create multifunctional platforms for dental applications. This review provides a comprehensive survey of and information on the status of this state-of-the-art technology and describes the development of glass ionomers reinforced with carbon nanotubes possessing improved mechanical properties. The applications of carbon nanotubes in drug delivery and tissue engineering for healing infections and lesions of the oral cavity are also described. The review concludes with a summary of the current status and presents a vision of future applications of carbon nanotubes in the practice of dentistry.

## 1. Introduction

The profound scientific, technological, and engineering impacts of the Fourth Industrial Revolution, also referred to as “Industry 4.0”, are being experienced across many aspects of society [1]. One series of outcomes relates to the dramatic changes occurring in the creation and conduct of research, with significant growth appearing in completely new areas, and the development of innovative research initiatives in underexplored areas to address specific societal and healthcare needs [2]. This ongoing evolutionary process is shifting many facets of human practices into a new era: the era of nanotechnology, the art and science of materials engineering at a scale of less than 100 nm [3]. “Nano” is a prefix derived from the ancient Greek word “nános”, which means “dwarf”. It refers to 1 billionth part of a particular physical size. Therefore, one nanometer represents 1 billionth (1 × 10^−9^) of a meter [4]. In the past 15 years, research on nanotechnology has revolutionized many diverse medical and dental research outcomes by seeking new strategies and more effective ways to apply the principles of bioengineering, cell-transplantation, and drug delivery to construct biological substitutes to maintain and restore the functions of diseased and compromised tissues [5].

The many applications of nanotechnology have led to significant improvements in healthcare in general medicine and in dentistry in particular. One aspect that has encouraged new advances in dentistry is the development and utilization of innovative nanomaterials in oral health practices [6]. As an example, biomimetic nanotechnology emulates the structure of dental enamel and the surrounding tissues to promote and achieve remineralization. The remineralization process focuses on restoring the amelogenin-based peptides, together with hydroxyapatite, to recover the hardness of the tooth [7]. These improvements in dentistry are represented by the development of a toothpaste comprising nanosized carbonate apatite, which effectively seals dentin tubules, promoting the management of dental sensitivity [8]. The aims of nanotechnology are to analyze structures, understand the physical properties, and create functional constructs through direct action on the existing framework [9]. Similar to nanomedicine, the use of nanotechnology in dentistry, known as “nanodentistry”, promotes the achievement of near-optimum oral health through the use of nanomaterials (including in tissue engineering and drug delivery systems) as integral approaches to dental restorations [10].

The aim of this review is to present relevant aspects regarding the use of nanotechnology in the development of dental restorative materials such as glass ionomer cements (GICs), and to discuss the influence of carbon nanotubes in drug delivery systems and tissue engineering for dental applications currently and in the future.

## 2. Carbon Nanotubes in Dentistry

Of all the elements in nature, carbon has the unique ability to exist in several different forms and structures, from carbon black to diamonds, with the resulting diverse applications. One of these scaffolds is the formation of nanotubes [11].

### 2.1. Characteristics of Carbon Nanotubes

Carbon nanotubes (CNTs) comprise a hollow, cylindrical structure of a hexagonal network of carbon atoms, measuring from several nanometers to a few microns. Various forms of this material can be produced using different techniques, including electric arc discharge, laser ablation, catalytic decomposition of hydrocarbons, electrolysis, synthesis from bulk polymers, and low-temperature solid pyrolysis, among others [12]. Carbon atoms in nanotubes are exclusively sp^2^-hybridized and are arranged in a hexagonal lattice. The resulting material has a high surface area, a small diameter, and high curvature. These characteristics contribute to their many unique properties through van der Walls, π-π stacking, and hydrophobic interactions. CNTs can be modified to increase solubility and modulate the inclusion of functional groups for specific biomedical applications [13].

In the past two decades, significant research has examined the development and application of carbon nanotubes in dentistry due to the mechanical (among other) properties that they exhibit. In this aspect, they are excellent candidates to act as a reinforcement for dental materials, for use as scaffolds, and for targeted drug delivery systems [14]. The ability to modulate these properties enhances the utility and the clinical performance of the nanocomposite material.

Despite these positive attributes, there is a limit to the loading of CNTs that can be added as a filler or reinforcement to polymers or other materials. This phenomenon occurs because of the agglomeration of CNTs. This is a major challenge in their use and is usually addressed through chemical functionalization which increases dispersion of CNTs and consequently leads to lower agglomeration. This effectively provides a higher loading capacity for the matrix, with benefits regarding the enhanced mechanical properties of the final composite [15]. Loading optimization for CNTs in the composite matrix is carried out with the aim of maximizing the efficiency of stress transfer to the matrix interface of the CNT. Variables such as interfacial stress transfer, aspect ratio, dispersion, and alignment, as well as the type of synthesis, modulate the effectiveness of the reinforcement [16].

### 2.2. Single-Wall and Multiple-Wall Carbon Nanotubes 

**Figure 1:**
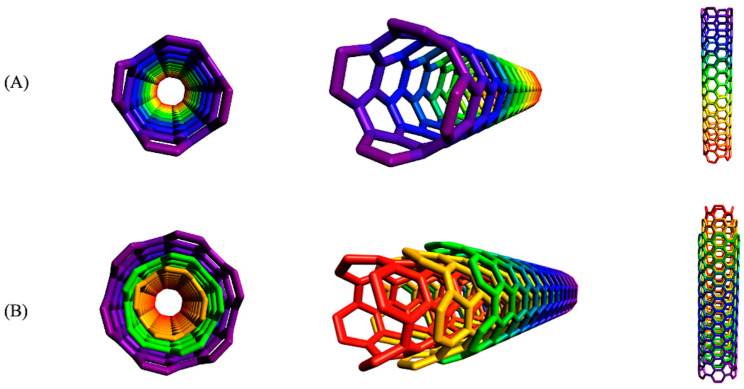
The 2 arrangements of the carbon atoms in (**A**) SWCNTs and (**B**) MWCNTs.

Both single- and multi-walled CNTs are available. Single-walled carbon nanotubes (SWCNTs) have a diameter between 0.4 and 2 nm, while that of multi-walled carbon nanotubes (MWCNTs) can vary between 2 and 100 nm. Lengths can extend to several millimeters long, a characteristic which depends entirely on the method of synthesis [17]. Figure 1 shows the respective arrangements of the carbon atoms in SWCNTs and MWCNTs. The angle of graphene rolling produces carbon nanotubes in three different formats: the achiral chair type (two sides of the hexagon oriented perpendicular to the axis of the CNT), the achiral zigzag type (two sides of the hexagon oriented parallel the axis of the CNT), and the chiral type (any pair of sides of the hexagon oriented at an angle different from 0 or 90° to the axis of the CNT). The different synthetic approaches do not mitigate the prevalent possibility of defects appearing in the nanotubes, such as Stone–Wales defects (90° change in π-π orientation), a pair of 5–7 rings, sp^3^-hybridized defects, and vacancies (absence of a carbon atom in the six membered rings) [18].

### 2.3. Applications of Carbon Nanotubes in Dentistry 

The specific applications of CNTs and their versatility depend on their individual properties, including morphology, size, and arrangement. In consequence, MWCNTs have emerged as promising candidates for delivery systems due to the high loading capacity provided by their greater surface area and their ability to interact with cellular membranes [19]. Additionally, their excellent mechanical and electrical properties indicate them as good candidates for use as fillers and as osteogenic scaffolds for bone proliferation and bone formation [20]. Indeed, carbon nanotubes used as fillers exhibited good performance in reinforcement at concentrations of 10 vol%, due to the load transfer on the interfacial bonding of the reinforced material and the CNTs [21]. 

The utilization of endogenous bioactive materials stimulates a diversity of biological interactions and elicits a cascade of responses from the living tissue [22]. To be effective in the oral cavity, a bioactive material must show characteristics which embrace the control of microbial infection, the strengthening of teeth, and have bio-promoting effects. These include control of inflammation, remineralization of teeth, and/or promotion of tissue regeneration [23]. In dentistry, these exogenous materials can be used in permanent restorations, for example as pulp-capping materials, for dentinal tubule occlusion, for scaffolding, and to promote tooth remineralization [24]. One example of bioactivity is the remineralization of initial caries lesions, where the dentin and enamel beneath the restoration can benefit significantly from the promotion of biomineralization, providing adhesion is not compromised [25]. 

## 3. Dental Restorative Materials 

The lifespan of dental restorations is limited and inherently depends on different factors, including the type of dentition (primary or permanent teeth), size, site, function of the restoration, and the composition of the dental material [26]. With recent scientific advances and an enhanced understanding of the caries process, minimal intervention procedures are performed to maintain the majority of the natural tissue, thereby providing increased life expectancy of the restoration [27]. 

One of the most important goals in contemporary dentistry is to reduce the failure rate of dental restorations due to bulk/marginal fractures and ameliorate the risk of secondary caries, thereby minimizing the need for a replacement restoration, with the consequent further destruction of tooth tissues [28]. Secondary caries represents one of the main issues leading to the accumulation of biofilm and consequently restoration failure [29]. Some authors claim that the rate of secondary caries in dental restorations may be as high as 50–60% due to the lack of resistance and adhesion of the dental material to tooth tissues, producing microleakage and promoting a high level of deleterious microbial activity [30]. 

Several materials have been introduced in dentistry as fillers and restorative materials to improve the treatment of both carious and non-carious lesions. With the evolution of dentistry and the emphasis on personal oral care practices, patients have a greater desire to maintain their natural teeth, which leads to an increase in the rate of dental restorations [31]. Restorative dental materials must embrace three fundamental characteristics to deal with tissue failure: (i) adhesion to the tooth tissues, (ii) appropriate mechanical properties, similar to dentin and enamel, and (iii) a wide range of color options [32]. New synthetic materials must overcome the challenge of the continuously moist environment of the oral cavity, and must withstand the effects of masticatory forces, variation of temperature, pH, microbial and enzymatic attacks, and be resistant to color changes from the exogenous materials present in foods (chlorophylls, anthocyanins, carotenoids, synthetic dyes, etc.). Bite forces may vary depending on the tooth location and the particular individual, for which values are in the range of 100 to 500 Newtons [33,34]. 

**Figure 2:**
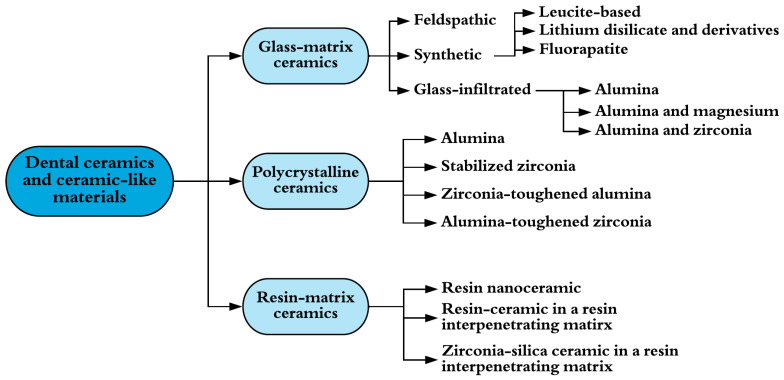
Classification system of ceramic and ceramic-like materials used in dentistry. Adapted from [38].

Traditionally, dental amalgam has been used for dental restoration [35]. This material is formed through the reaction of a powdered alloy, composed of silver, tin, and copper, with mercury. The resulting malleable mass is used as a dental restorative. Despite its history of use in the restoration of posterior teeth, this material suffers from several negative characteristics. The most important one is that this material does not adhere to the dental tissues and requires the sacrifice of caries-free tissue to provide the required mechanical retention [36]. Toxicity for humans, the potential for environmental contamination, and esthetic issues have increased concerns with the continued use of this material, leading to research developments for alternative restorative materials [37]. Ceramic materials have found wide use in restorative dentistry and are classified into three groups: glass-matrix ceramics, polycrystalline ceramics, and resin-matrix ceramics (Figure 2), as proposed by Gracis et al. [38]. 

In addition to these materials, there are several dental restorative materials which employ resin composites. These were developed to replace lost or decayed tooth structure, attending to demands for better esthetic appearance and minimizing the hazards of using mercury in dental amalgams. Although these resin-based composites are widely used for restorations due to their color similarity to natural teeth, and for cavities subjected to low level stress [39,40,41], their use is limited due to issues with mechanical strength, wear resistance, polymerization shrinkage, and color stability. The main benefit in comparison with other materials is the cost of the preparation, which makes them more affordable for patients [42]. 

### 3.1. Characteristics of Glass Ionomer Cements 

Glass ionomer cements (GICs) are dental materials that display attractive properties for use as restorative and luting materials. They were introduced in 1972 by Wilson and Kent as a “new translucent dental filling material” and consist of three basic components: a polymeric water-soluble acid, a basic (ion-leachable) glass, and water [14]. This type of material produces different properties from a powdered fluoroaminosilicate, such as strength, rigidity, and fluoride release. It also takes advantage of the biocompatibility and adhesive characteristics of the polyalkenoic acid component [38,42]. Glass ionomer cements can be classified as shown in Table 1. 

**Figure 3:**
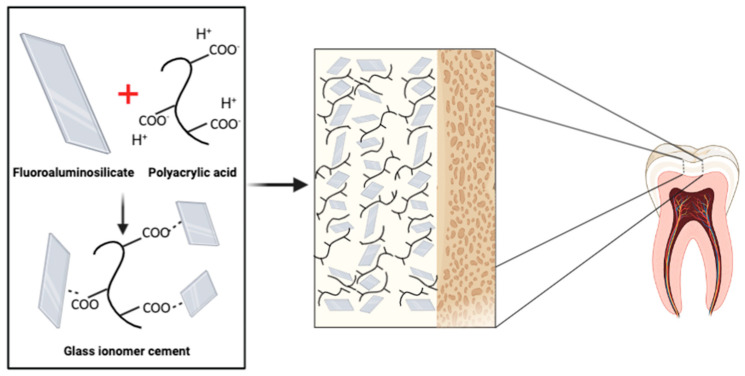
Basic composition of a glass ionomer cement (GIC) and its interaction with a tooth.

Glass ionomer cements (GICs) are formed as the result of an acid–base reaction. The acid attacks and degrades the alumina-silicate glass, releasing cations of calcium and aluminum. Chelation of cations occurs between the carboxylate groups and the polyalkenoic acid chains form a cross-linked structure (Figure 3) [14]. 

The mechanical resistance of the GIC material is a key factor to provide good performance of restoration for the patient. This requires consideration of the composition and mechanical properties of the natural tooth since enamel and dentin have different mechanical properties (Table 2). It was reported that the coefficient of thermal expansion of GICs is similar to that of human dentin, thereby reducing the possibility of marginal leakage and restoration failure [50]. 

### 3.2. Benefits and Applications of Glass Ionomers in Dental Restorations 

Currently, GICs are widely used in several dental applications, including full dental restorations, fissure sealants, luting agents, liners, and bases, and as endodontic sealers due their unique properties in comparison with other conventional materials [52]. These properties include chemical adhesion to enamel and dentin in the presence of wetness, high biocompatibility, resistance to microleakage, favorable thermal expansion and contraction, good marginal integrity, fluoride release, and stability at high humidity [53]. 

The need for replacement restorations typically arises due to the development of secondary caries at the interface of a restoration and tooth tissue. Bacteria present in the human oral micro-environment, including *Streptococcus mutans*, *Actinomyces* spp., and *Lactobacillus* spp., are the main microorganisms responsible for development of dental biofilm [14]. In addition, inherent surface unevenness of glass ionomers increases the surface area and provides niches for biofilm formation. GICs are considered to be cariostatic and antibacterial materials due to fluoride release [28] and their strong bonding to tooth tissues. However, the physical properties of these materials limit their use for posterior tooth restoration [54]. An interesting strategy would be to develop dental materials with selective antimicrobial activity for dominant oral pathogens through the incorporation of nano-delivered natural compounds derived from plants or microorganisms. This could be especially impactful for the creation of novel restorative materials. 

### 3.3. Major Drawbacks of Using Glass Ionomers 

Even though the glass ionomer cements possess many attractive characteristics, they present significant disadvantages in practice, including poor hydrolytic stability, low flexural strength, poor fracture toughness, and limited durability [55]. In addition, there are clinical limitations, including prolonged setting reaction time, dehydration, and the rough texture, which can reduce the final mechanical properties of the restoration after setting [56]. To overcome these clinical constraints and provide enhanced performance for the patient, further improvements are required, particularly to enhance mechanical strength. The use of resin-modified GICs has been proposed as one approach to correct partially the composition issue, to enhance the physical properties of the material, and to reduce fluoride ion release. When this release does occur, mineralization of the tissue is reduced, and the probability of restoration failure is higher [57]. 

### 3.4. Nanotechnology in Glass Ionomers 

Research for new materials with improved clinical performance has been continuous and has led to achievements of nanotechnology in dental material manufacturing. These new materials are established as “nanobiomaterials” and exhibit enhanced properties and efficiency in comparison with formative bulk materials [58]. To overcome mechanical and biological constraints of GICs, several nanomaterials, including hydroxyapatite, silica, zirconia, graphene, and silver nanoparticles, have been incorporated into GICs. Introduction of nanohydroxyapatite and silica into GICs resulted in improvements in mechanical properties (hardness and compressive and flexural strength) and maintained a sustained fluoride release [59]. When alumina/zirconia and hydroxyapatite were added to the GIC, antibacterial activity and biocompatibility were increased [60]. Among various nanomaterials, graphene has been used to reduce biofilm formation and to increase the wear resistance of dental composites [11]. 

The influence of multi-walled carbon nanotubes (MWCNTs) in reinforced glass ionomer cements with respect to their chemical, thermal, and mechanical properties for specific use as a posterior restorative material was examined by Goyal et al. [61]. Concentrations of 0%, 1%, and 2% *w*/*w* of MWCNTs were added as a reinforcement agent, obtaining a dark-colored material, which limits its application only to posterior teeth. An enhancement of mechanical properties of the material was reported as a hardness increase from 2.19 MPa to 5.70 MPa with the addition of 2% *w*/*w* of MWCNTs. This particular composition also tolerated higher wear forces better than the other two compositions [61]. 

The esthetic appearance of restorative materials is very important for all patients and is why GICs are widely used in the restoration of primary teeth. Color stability tests must therefore be conducted on new materials to determine if they will meet the requirements of the individual patient. The incorporation of carbon nanotubes into glass ionomer cements has led to enhanced color stability profiles compared with other reinforcement materials such as silver nanoparticles [62]. Based on these considerations of color stability this material can be used effectively in posterior restorations, especially for primary teeth where esthetic requirements and color stability are less clinically significant [63]. 

## 4. Carbon Nanotubes in Guided Bone Regeneration (GBR) 

Currently evolving targets are shifting towards the development of biofunctional materials which prevent disease and/or actively promote tissue regeneration. In the instance of a bone defect, guided bone regeneration (GBR) is now a widely used technique which deploys an occlusive membrane to seal the area of the bone defect to physically prevent the incursion of non-osteogenic cells into defects [64]. This artificial barrier also serves as a scaffold which fosters osteogenic cells to stimulate bone formation at a higher rate than the surrounding connective tissue and prevents infection when used in dental implants. Requirements for an ideal material used in GBR include: (i) biodegradability; (ii) biocompatibility to promote integration with the tissue and avoid inflammatory responses; (iii) mechanical strength; and (iv) porosity to be partially occlusive to avoid epithelial cell flow and fibroblast of the soft tissue, and allow the diffusion of oxygen, nutrients, and bioactive substances [65].

Innovation of artificial scaffold materials to sustain bone cell proliferation and growth, and to enhance incrementally or replace bone tissue, is a primary goal in bone bioengineering [66]. In support of this biological and clinical outcome, CNTs have been explored for the stimulation of bone regeneration and to provide an alternative permanent mechanical function. It was observed that neutrally charged CNTs have the ability to sustain both osteoblast proliferation and bone forming functions, and they showed promising biocompatibility with osteoblast cells [67].

Research studying the effects of the surface functionalization of CNTs in nanocomposites for dental implants, such as hydroxyapatite, zirconia, and titanium, has fostered a greater understanding of the chemical groups that can promote osteoblast proliferation [68]. In addition, carbon nanotubes combined with hydroxyapatite demonstrated cytocompatibility, with more than 200% cell viability and compressive strength in the range of 13 to 29 MPa. This combination is considered an attractive bone-filling material [69]. 

### 4.1. Mechanical Properties of Nanofiber Polymeric Membranes Reinforced with Carbon Nanotubes 

Microcracks in bone are the natural response of the tissue to excessive applied mechanical loads. With the introduction of carbon nanotubes as scaffolds the reinforced bone tissue is more resistant to the development and growth of fissures due to better load distribution, dispersal of the crack growth, and a decrease in the stress intensity near the fissure tip [70]. The addition of different functional groups, including amino, phosphate, and carboxylic acid moieties, can dramatically change the physical, mechanical, and biological properties of CNTs with respect to bone growth. This functionalization will depend entirely on the specific needs of the load (drugs, antigens, genes, etc.). Functionalization also assists in CNT biodegradability through the introduction of structural defects which leads to the improved intrusion of oxidative enzymes to enhance the degradation of CNTs [71]. Interaction of carbon nanotubes with tissue environment is modulated by tailoring the functional groups at the surface, as this determines the charge density and the overall net polarity. The result is that a charged surface obtained through chemical modification may be more hydrophilic, in contrast to the initial, electrically neutral surface [68]. 

The use of CNTs as fillers in the reinforcement of scaffold materials is affected by four parameters: (i) their extent of dispersion in the matrix, (ii) their aspect ratio, (iii) their alignment, and (iv) the interfacial stress transfer. Their distribution allows the material to have a uniform performance and higher superficial area, while the alignment of the nanotubes is reflected in their improved mechanical strength, and stress transfer allows the matrix to carry higher loads without cracking [16]. Maximization of the load transfer is achieved by incrementing towards a larger aspect ratio. Measurement of these improvements is possible using microindentation, which consists of applying a specific force using a diamond indenter to measure the declinations of enamel rods and dental tubules [72]. The interaction between the polymer and the CNT is particularly important in order to transfer the external stress forces to the CNTs, thereby enabling the matrix to bear higher loads [73]. 

In addition to this application, the use of these materials for dentin surface modification showed selective coating of the surface of the dentin and cementum by adhering to the collagen fibers exposed from these surfaces [74]. Hahn et al. reported the ability of carbon nanotubes to improve mechanical properties, such as hardness and elastic modulus, when CNTs are added to a hydroxyapatite coating, resulting in an adhesion strength ranging from 27.3 to 29.0 MPa [75]. Similarly, Marrs et al. studied the application of CNTs for the reinforcement of a bone cement based on polymethylmethacrylate (PMMA). The result was an enhancement in mechanical properties with peaks of performance observed with concentrations of 2% wt. The product showed a flexural strength of 90.6 MPa and a bending modulus of 3528 MPa [76]. Bonding, esthetic, mechanical, and physical properties of restorative dental materials have been greatly improved. Although these materials show excellent clinical response, investigations to achieve performance similar to the attributes of natural teeth continue as an ongoing process [77]. 

Scaffolds for bone tissue engineering applications containing polycaprolactone (PCL) have been widely used. PCL is a hydrophobic semicrystalline polymer whose crystallinity decreases with the increment of molecular weight. This material has good solubility and a low melting point (59–64 °C) [78]. It is a biodegradable and bioactive material and has been considered as a possible substitute for bone tissue due to its unique properties. However, the application of PCL for this purpose is limited by its weak mechanical properties. This disadvantage can be addressed through the production of polymeric nanofiber composites formed through electrospinning processes [79]. The resulting polymer is easily synthesized and processed and can be molded precisely into diverse shapes which are easily modified due to the viscoelastic properties and the low melting temperature. A clinically important characteristic of modified and electrospun PCL is that it can be functionalized with active molecules such as drugs or bone growth stimulating factors [80]. Previous studies have also shown that PCL reinforced with CNTs exhibited improved physicochemical, biological, and mechanical properties, for example through the increment in elastic modulus and tensile stress as well as conductivity, while they did not exhibit cytotoxicity [70,71].

### 4.2. Effect of Carbon Nanotubes on Cells

The cytotoxicity of CNTs has been widely studied. It was demonstrated that the cytotoxicity of CNTs depends on the relationship with length, diameter, and the presence of functional groups. To mitigate toxicity, many efforts have been made to modify the surface properties of CNTs. For instance, Ketabi et al. studied the reinforcement of nanofibers of polycaprolactone with multiple-wall carbon nanotubes for odontoblast cell interactions [81]. In the same way, Flores-Cedillo et al. reinforced polycaprolactone with MWCNTs for use as scaffolds in bone tissue regeneration, thereby developing materials with improved physicochemical, biological, and mechanical properties, such as increased elastic modulus and tensile stress, as well as conductivity. While they did not exhibit cytotoxicity, it was concluded that the application of an electric field to the carbon nanotubes does not promote alignment or dispersion [42]. 

The diameter of carbon nanotubes provides them the capacity to inactivate bacteria such as *E. coli.* Direct contact of the bacteria with the material causes cell membrane damage and subsequently cell death. Differences in toxicity lie in the diameter of the tube and the surface area available for interaction. Kang et al. demonstrated that single-wall CNTs were more toxic for bacteria than multi-walled CNTs through oxidative stress. To quantify the molecular response of the bacteria with the CNT gene expression, DNA microarray analyses were performed [82]. 

A recent study investigated the influence of coating biopolymer nanofibers with CNTs on cells [83]. It was demonstrated that these scaffolds could modulate different interactions of the cells and tissues that allow bone healing and regeneration, reduce inflammatory signals, and promote angiogenesis. The in vitro study demonstrated accelerated adhesion and osteogenic differentiation, while the in vivo results showed an increase of bone forming cells with higher bone mineral density, confirming the potential use in bone regeneration and healing processes [83]. 

### 4.3. Preparation of Polymeric Membranes Reinforced with Carbon Nanotubes 

**Figure 4:**
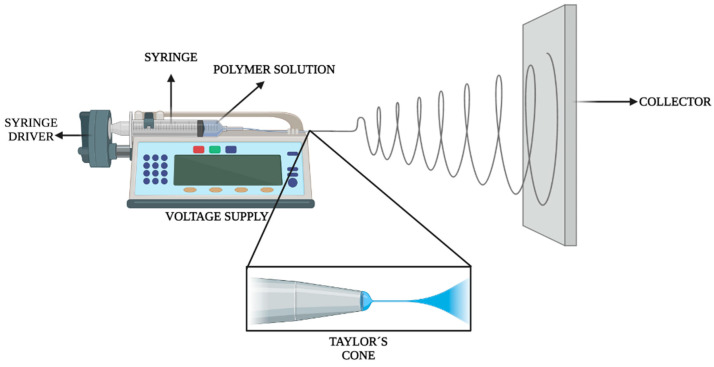
Illustration of the electrospinning method.

The most common method to prepare polymeric membranes is through electrospinning, a process which was introduced in the 1930s to produce polymeric fibers from several nanometers to a few micrometers [84]. Electrospinning, as illustrated in Figure 4, is based on the dispersion of a polymer in an appropriate solvent, which is then introduced into a glass syringe. The polymeric solution is pumped through the syringe to which is applied a high voltage (25 kV, positive pole), while the collector acts as the negative pole. Due to the voltage difference that exists between the tip of the needle and the collector, the polymeric material becomes stretched thereby forming a Taylor´s cone, which provides polymeric fibers ranging from 30 nm to 1 μm in diameter [85]. The resulting membranes can be used for many different applications, including fine filtration, scaffolds for tissue engineering, and drug delivery systems. 

The synthesis and characterization of nylon-6 fibers using electrospinning, with and without the reinforcement of carbon nanotubes, demonstrated the significant influence of CNTs and how the concentration of CNTs (2.5, 5.0, 10.0, and 20.0%) affected mechanical properties such as flexural strength, volumetric polymerization shrinkage, and elastic modulus, with the best values obtained for concentrations of 2.5 and 5.0% of CNTs. The highest value for flexural strength was reported as 106.0 MPa for a 5% concentration with an elastic modulus of 201.0 MPa [86]. Polystyrene/MWCNT nano fibers showed an increase in Young’s modulus of 22% compared with untreated polystyrene [87]. The preparation of nanofibers of polyurethane/MWCNTs showed an enhancement in the tensile strength compared with the bulk material [80]. Strength of polymeric nanofibers prepared through electrospinning is due to the differently adopted conformations of the polymer chains and the microstructure and is enhanced through interfacial linking forces between matrix and nanofillers, mainly due to the specific surface area of the nanofibers [88].

## 5. Carbon Nanotubes in Drug Delivery Systems 

Modifications in drug delivery systems have a profound effect on the bioavailability and pharmacokinetics of medicinal agents and represent a widely researched area of drug development. Currently, studies are taking advantage of the special properties of carbon nanotubes. Major improvements in targeted drug delivery systems include a reduction in drug dosage, retiming of drug distribution to obtain the same results, and reducing the side effects from the current delivery methods [89]. Carbon nanotubes provide an opportunity to introduce a high loading capacity and the ability to be easily taken up by the cells. It has been stated that a carbon nanotube with a diameter of 80 nm can accommodate approximately 5 million drug molecules, thereby by serving as a nanocontainer [90].

Although there are many available delivery systems their success is limited due to low protein loading, size control, and toxicity; carbon nanotubes are studied for use in biological systems based on their ability to penetrate cell membranes, their sustained capacity, and their distribution within cells [19]. MWCNTs have the capacity for high protein loading and stability under biological conditions [19].

The commonly suggested mechanism of interaction between carbon nanotubes and cellular membranes is through receptor-mediated endocytosis. The uptake mechanism arises from surface interactions of the media with the carbon nanotubes, an interaction regarded as the most important for cell internalization [91]. 

## 6. Conclusions and Future Applications 

This review demonstrates the highly successful impact of carbon nanotubes in dentistry. The continuous development of new materials for oral applications, including for functional dental restorations, demands the provision of new composites with enhanced physical, chemical, and mechanical properties to deal with the clinical needs prevalent in the oral cavity. Furthermore, dental composites are expected to exhibit a wide range of desired characteristics, including biocompatibility, adhesion to tooth tissues, and color stability, as well as the delivery of biological agents for prevention and treatment. 

Because the physicochemical and mechanical properties of carbon nanotubes are tunable, it is anticipated that their incorporation into dental materials will increase their use in dentistry, leading to new and more effective functional applications. These potential advancements could lead to the development of new materials for caries prevention with the use of functionalized carbon nanotubes as drug delivery systems, as well as biomimetic scaffolds that imitate the extracellular matrix for tissue engineering. In addition, carbon nanotubes hold great potential for the development of dental materials with interesting properties, including bioactivity, as a delivery system for agents with antimicrobial and tissue-regenerative properties. Undoubtedly, they will provide innovative platforms for a wide range of future studies which will improve oral care.

## Figures and Tables

**Table 1:** Classification and characteristics of the glass ionomer cements.

Classification	Characteristics and Use	Reference
Type I	Luting cements with low fill thickness and rapid setting. Used for the cementation of inlays, crowns, fixed partial dentures, and orthodontic appliances.	[43]
Type II	Restorations with particles larger than Type I.	[43]
Type II-1	Considered as esthetic cements available for conventional and resin-modified presentations.	[43]
Type II-2	Reinforced cement for esthetic applications.	[43]
Type III	Lining cements and fissure sealants with low viscosity and rapid setting.	[43]
Based on composition	Derived from an organic acid and a glass component referred to as acid-base reaction cements.	[44]
Resin-modified GICs	Contains an ion-leachable glass, a water-soluble polymeric acid, an organic monomer, and an initiator system.	[45]
Polyacid-modified composite resin	Light-polymerized composite resin restoratives with ion-leachable glass particles and an anhydrous polyalkenoic acid.	[46]
Metal-reinforced GICs	Mixture of a conventional powder with the addition of a range of metallic powders, such as silver alloys, gold, palladium, and titanium dioxide.	[47]
High-viscosity GICs	Have a high powder–liquid ratio and fast setting properties.	[48]
Zirconia-reinforced GICs	Contain zirconium oxide, glass powder, tartaric acid (1–10%), polyacrylic acid (20–50%), and deionized water.	[49]

**Table 2:** Mechanical properties of enamel and dentin. Retrieved from [51].

Tooth Tissue	Property	Value
	Hardness	2.0–3.5 GPa *
Enamel	Young’s modulus	80–120 GPa
	Fracture toughness	0.67–3.93 MPa m^1/2^
	Hardness	0.3–0.7 GPa
Dentin	Young´s modulus	10–40 GPa
	Fracture toughness	1.1–2.3 MPa m^1/2^ **

* GPa indicates gigapascals; ** MPa indicates megapascals. Fracture toughness is expressed in units of stress times the square root of crack length: MPa m^1/2^.

## Data Availability

Not applicable.
